# Implementation strategy and cost of Mozambique’s HPV vaccine demonstration project

**DOI:** 10.1186/s12889-019-7793-y

**Published:** 2019-10-29

**Authors:** Caroline Soi, Joseph B. Babigumira, Baltazar Chilundo, Vasco Muchanga, Luisa Matsinhe, Sarah Gimbel, Orvalho Augusto, Kenneth Sherr

**Affiliations:** 1 Department of Global Health, Harris Hydraulics Laboratory, University of Washington, 1510 San Juan Road, Seattle, WA 98195 USA; 2 Health Alliance International, 1107 NE 45TH St #350, Seattle, WA 98105 USA; 3 Universidade Eduardo Mondlane, Av. Salvador Allende no. 702, Maputo, Mozambique; 4 Health Alliance International, Rua Caetano Viegas no. 67, Maputo, Mozambique; 5 Department of Family and Child Nursing, University of Washington, Magnuson Health Sciences Building, 1959 NE Pacific St, Seattle, WA 98195 USA

**Keywords:** HPV vaccine, Cost, Implementation strategy, LMIC, Mozambique, Demonstration project

## Abstract

**Background:**

Cost is an important determinant of health program implementation. In this study, we conducted a comprehensive evaluation of the implementation strategy of Mozambique’s school-based HPV vaccine demonstration project. We sought to estimate the total costs for the program, cost per fully immunized girl (FIG), and compute projections for the total cost of implementing a similar national level vaccination program.

**Methods:**

We collected primary data through document review, participatory observation, and key informant interviews at all levels of the national health system and Ministry of Education. We used a combination of micro-costing methods—identification and measurement of resource quantities and valuation by application of unit costs, and gross costing—for consideration of resource bundles as they apply to the number of vaccinated girls. We extrapolated the cost per FIG to the HPV-vaccine-eligible population of Mozambique, to demonstrate the projected total annual cost for two scenarios of a similarly executed HPV vaccine program.

**Results:**

The total cost of the Mozambique HPV vaccine demonstration project was $523,602. The mean cost per FIG was $72 (Credibility Intervals (CI): $62 - $83) in year one, $38 (CI: $37 - $40) in year two, and $54 CI: $49 - $61) for years one and two. The mean cost per FIG with the third HPV vaccine dose excluded from consideration was $60 (CI: $50 - $72) in year one, $38 (CI: $31 - $46) in year two, and $48 (CI: $42 - $55) for years one and two. The mean cost per FIG when only one HPV vaccine dose is considered was $30 (CI: $27 - $33)) in year one, $19 (CI: $15–$23) in year two, and $24 (CI: $22–$27) for both years. The projected annual cost of a two-and one-dose vaccine program targeting all 10-year-old girls in the country was $18.2 m (CI: $15.9 m - $20.7 m) and $9 m (CI: $8 m - $10 m) respectively.

**Conclusion:**

National adaptation and scale-up of Mozambique’s school-based HPV vaccine strategy may result in substantial costs depending on dosing. For sustainability, stakeholders will need to negotiate vaccine price and achieve higher efficiency in startup activities and demand creation.

## Contributions to the literature

- Research has shown considerable heterogeneity within and between countries for HPV vaccine delivery cost determinants. Our findings provide country specific cost data that can inform Mozambique’s national immunization program HPV vaccine delivery scaling up strategy
- The paper provides a costing framework and methodology that other HPV vaccine delivery researchers can replicate, thus promoting generalizability of research findings
- Our findings contribute to identified gaps in costing literature for delivery of HPV vaccines in low- and middle-income countries

## Background

Cost is an important determinant of successful implementation of healthcare programs [1, 2]. Health program implementation is associated with costs over and above the costs of the intervention itself and exclusion of implementation costs may bias economic evaluation results [3]. Costs are incurred during different phases of implementation and measuring and documenting implementation-phase-specific costs may provide useful information for national policy makers and health program managers engaged in decision making for adoption of health service innovations [4]. Such cost information is also useful to researchers attempting to standardize implementation research costing methods and produce generalizable study findings [5].

The human papilloma virus (HPV) is a common sexually transmitted organism that infects the anogenital and airway tracts where it may remain asymptomatic or manifest as warts, papillomatosis or cancer [6, 7]. Global HPV infections are associated with an estimated 50% of cancers in women and 5% in men, with varying burden depending on anatomical site. Anogenital sites are more significantly affected with proportions of more than 99% of cervical, 97% of anal, 70% of vaginal, 47% of penile and 40% of vulval cancer cases, compared to 47% of oropharynx and 11% of oral cavity cancer cases [8]. HPV types 16 and 18 account for more than 70% of invasive cervical carcinoma (ICC), the most significant cancer caused by the HPV [9]. Approximately 530,000 cases of cervical cancer occur in the world annually, with 50% progressing to death [10]. Low and middle-income countries (LMICs) in which screening programs and treatment options are limited, bear the brunt of the ICC burden with 85% of diagnosed cases and 87% of deaths [11].

The highest rates of age-standardized HPV incidence and mortality, 42.7 and 27.6 per 100,000 women respectively, are found in East Africa [12]. Mozambique has particularly high age standardized incidence rates (65.0 per 100,000) and mortality (49.2 per 100,000) and places second in the global ranking of cervical cancer burden [13]. Studies in Mozambique suggest that HPV is the main determinant of this large burden of cervical cancer: the prevalence of HPV, abnormal cervical cytology, and cervical neoplasia are as high as 40, 19, and 12% respectively, and HPV types 16 and 18 are present in 78% of biopsies from patients with cervical cancer [14].

Since its introduction in 2006, the HPV vaccine has demonstrated a high level of effectiveness for prevention of cervical cancer [15]. Three types of the vaccine exist: bivalent, quadrivalent and nonavalent. HPV vaccination has led to a decline in HPV-related disease in high-income countries [16–18]. The World Health Organization (WHO) recommends adoption of HPV vaccination in LMICs, such as Mozambique, where cervical cancer is a major public health priority [19]. Consequently in 2011, Gavi, the Vaccine Alliance (Gavi) added the HPV vaccine to its list of financing-eligible vaccines, paving the way for its inclusion in national immunization programs (NIPs) in LMICs [20]. However, a challenge was encountered: girls ages 9–13 years, the target group for HPV vaccination, are not routinely targeted for immunization and interface with the health system is limited because adolescent-specific health services are minimal to non-existent in these settings [21]. As such Gavi’s initial HPV vaccine introduction strategy required countries to conduct demonstration projects to test possible delivery modalities prior to national rollout [22]. Many LMICs leveraged this opportunity and to date, 30 countries have conducted demonstration projects [23]. However, progress to national scale up of HPV vaccination has been slow: only six (of 30) countries had commenced national HPV vaccination programs by December 2017 [24, 25]. One barrier to national scale up of HPV vaccination programs is the higher delivery costs associated with setting up a new delivery model for the 9–13 year old target age group [26]. Nevertheless, current strategies to improve vaccination uptake [27, 28] coupled with evidence of long-term benefits from vaccine investment [29, 30] provide a strong rationale for allocation of resources to HPV vaccination programs which seem relatively expensive.

A recent review of the costs of introduction of HPV vaccines in LMICs concluded that country-specific HPV cost data were lacking and that there was a need for more information from country demonstration projects [31]. The aim of this study was to contribute to filling this gap in knowledge by conducting an in-depth analysis of the implementation costs of the demonstration project that was conducted in Mozambique. Specifically, we sought to estimate the cost of a fully immunized girl (FIG) and the costs of rolling out a similar program nationally.

## Methods

We collected implementation cost data between January 2014 and March 2017 as part of a process evaluation of the Mozambique HPV demonstration project. The specific methods we utilized were: document review, participatory observation, and key informant interviews (KIIs) Additional file 1.

### Implementation costing approach

Our study was performed from the payer perspective, in this case the Government of Mozambique through the Ministry of Health [32]. The micro-costing approach followed the three steps of *identification, measurement* and *valuation* [33]. The resource items necessary for HPV vaccination were identified using the principle of the production function of a health care program [34]. We supplemented primary data with data from WHO guidelines for estimating costs of introducing new vaccines into NIPs [35] and guidelines for HPV scale up [36], as well as published literature on HPV vaccine costing [37, 38].

Costs were divided into direct medical and direct non-medical costs [39]. Resources were identified and measured using data from HPV demonstration project documents and the comprehensive multiyear plan (cMYP) of the Mozambique NIP [40]. These data were supplemented with participatory observations and KIIs conducted as part of the demonstration’s project process evaluation. Gross costing methods were used to analyze data obtained in bundles from the Mozambique Ministries of Health (MOH) and Education for salaries, travel allowances, fuel and other activities implemented by the NIP [37].

### Delivery strategy for the HPV vaccine demonstration project

**Fig. 1:**
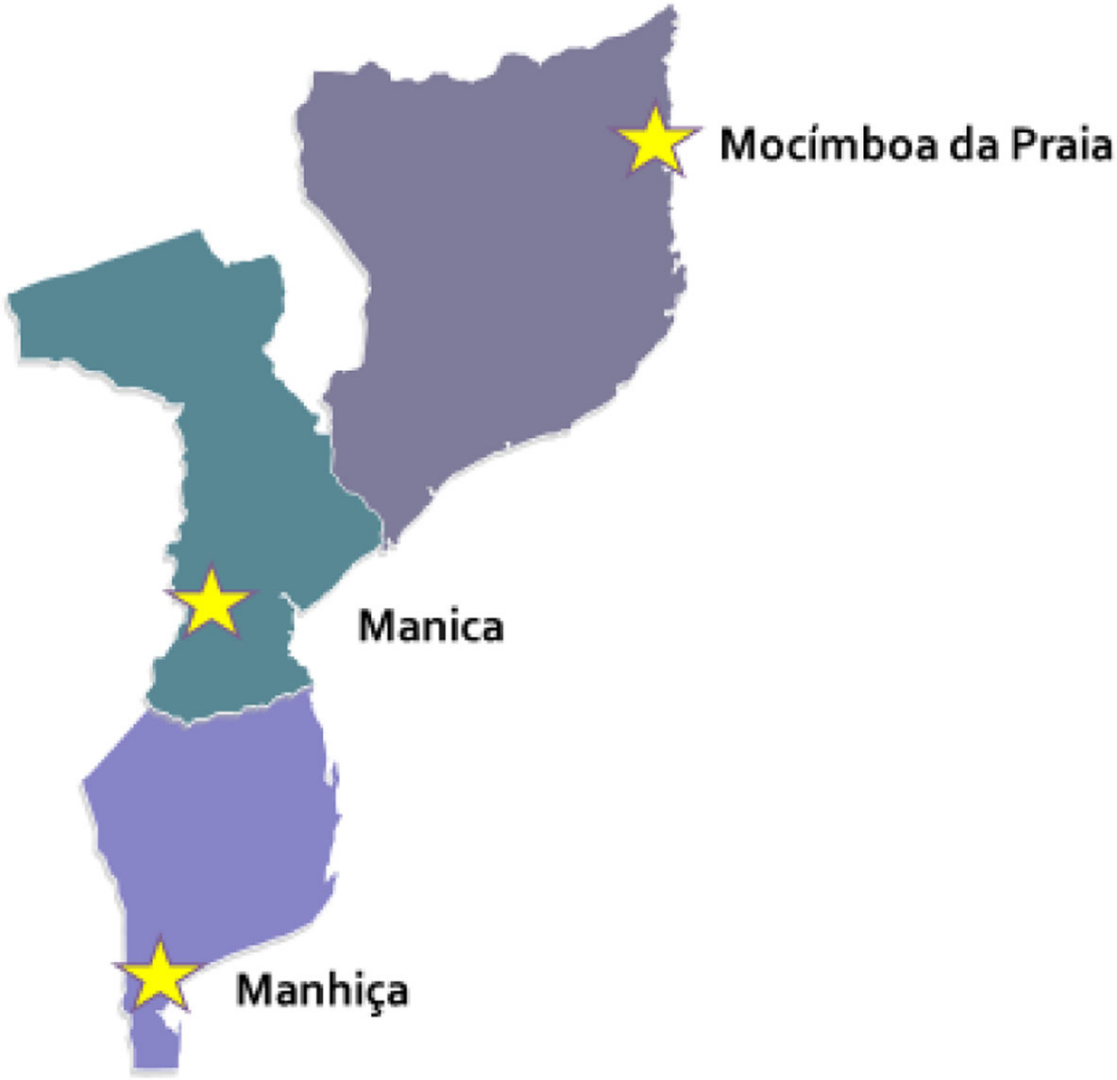
Mozambique HPV vaccine demonstration project sites. Source: https://yourfreetemplates.com/free-mozambique-editable-map/

The HPV vaccine demonstration project was conducted in three districts: Manhiça, Manica and Mocímboa da Praia. The districts were selected to reflect the three socioeconomically diverse regions of the country: south, central and northern (Fig. 1).

The government received Gavi funding for only Manhiça district, and consequently more vaccine-related activities were performed in this district relative to the other two.

The demonstration project was school-based. Vaccines were delivered over 1 week with subsequent community outreach visits in the days after the school-based vaccinations. In order to simplify the identification and vaccination of adolescent girls, the 10-year age group was selected because they were more likely to be found in “a single class” at schools, enabling targeting to avoid the disruption that would occur if multiple classes were targeted. In the first year of the project, each eligible girl received three doses of the bivalent Cervarix™ vaccine, administered one each in May, June, and November in order to fulfill the WHO recommended schedule of 0, 1, and 6 months. In the second year, following revised WHO guidelines, the number of doses was reduced to two, scheduled at 0 and 6 months. These were administered in June and November to a new group of eligible 10-year-old girls.

Teams composed of a health worker and an auxiliary staff member from all health facilities in the district made visits to up to two community spots (in addition to schools) during the course of the five weekdays of the vaccination week. The teams spent about 6 h in each school and 6 h in each community outreach spot. In each school, teams identified a teacher to arrange a vaccination venue, register the girls prior to the vaccination day, and organize them in queues ready for vaccine administration by the health workers. The same teacher observed the girls for any symptoms of adverse effects after immunization (AEFI) for a period of up to 48 h. Overall supervision was provided by two provincial and two district supervisors for each of the three demonstration districts. Additionally, two supervisors from the national level visited each district during the vaccination week to provide oversight and technical support.

Preparatory activities that were undertaken prior to vaccination included vaccine procurement, social mobilization, training, and development and revision of monitoring tools. Vaccines and injection materials were procured and distributed through the existing national immunization cold chain system from the national warehouse in the national capital Maputo to health facilities in the three demonstration districts. Social mobilization messages were developed, piloted and then finalized. Different types of information, education and communication (IEC) materials were developed and produced, including brochures, and radio and television media spots. Additionally, community leaders were engaged to provide community talks in all districts and community activists made door to door visits in only the one district that received Gavi funding. A call-in educational service was started to enable community members to call in and ten telephone operators were trained and provided services during the demonstration project. The three districts each conducted a launching ceremony in which costs were incurred for promotional material items such as banners, T-shirts and caps. Training was conducted in a cascade manner for the health workers and Ministry of Education staff. Monitoring tools that were developed and printed included tally sheets, registration books and vaccination cards. A post introduction evaluation was carried out in Manhiça district where the Ministry of Health received financial support from Gavi.

### Base-case cost assumptions

Given that HPV vaccines were delivered to schools and communities where eligible girls were located, recipients and their caregivers did not incur any costs to seek vaccination services. As such patient costs were not considered.

The direct medical costs comprised procurement of vaccines and vaccine injection supplies, vaccine distribution, cold chain costs, social mobilization, personnel, outreach visits, and monitoring and evaluation (M&E). The total annual vaccine cost included: (1) the cost per vaccine dose ($5.70 (including shipping costs)), (2) vaccine coverage for years one and two (number of girls vaccinated), (3) number of doses per girl (three in year 1 and two in year 2), (4) the observed wastage rate of 5%, and (5) a buffer stock of 25%. Vaccines were procured at the beginning of the two-year demonstration period. Vaccine injection supplies which were procured together with the vaccines included syringes and safety boxes. Syringe quantities were assumed to equal the vaccine doses while each five-liter safety box can hold 100 syringes. The syringe wastage rate used was the standard 10% utilized by the Mozambique NIP.

Vaccine distribution costs were related to air and road transport. Cold chain costs included storage costs calculated for the period that the HPV vaccines were stored prior to their utilization. Vaccines arrived in- country 6 months prior to the vaccination date and required storage until the last dose had been administered in the second year. The cold chain volume in liters per month was estimated and multiplied by the unit cost (cost per liter). We took into account the decrease in vaccine volume over the months, as well as the amounts of time the vaccines spent at national, provincial, district and health facility storage facilities. HPV vaccines did not require a different distribution timeline from other NIP vaccines. Their distribution was integrated into the routine monthly mechanism existing at all health system levels. For social mobilization, we obtained unit costs from project documents to compute the total costs for training and incentivizing community leaders and volunteers. Units costs were used for the calculations of all IEC materials while lump sum quantities were used for television and radio spots development, production and broadcasting.

Personnel costs included training costs and time health workers spent on outreach visits calculated in hours spent at schools and community outreach spots multiplied by wages per hour. Outreach visit costs comprised of health worker per diems and fuel. For monitoring and evaluation, we considered national, provincial and district supervisor per diems, air tickets for national level supervisors and fuel costs. The post introduction evaluation costs included per diem and fuel costs. Lump sum costs were utilized for the development and printing of monitoring tools. All fuel cost calculations are based on kilometers travelled and amount of fuel required in liters. Training costs comprised of travel per diems, training materials and venue costs and were based on actual values incurred during the demonstration project. Education workers’ costs included training costs and time spent during vaccination activities and post vaccination observations for adverse effects which were calculated in hours and multiplied by the wage cost per hour.

Direct non-medical costs were composed of overhead administrative costs. We estimated these by taking the overall annual provincial and district immunization program overhead costs and dividing them by the 52 weeks in a year. We then calculated costs needed during the 5 weeks that HPV vaccine was administered plus another 6 weeks for preparatory activities (2 weeks prior to the initial dose and 1 week for each of the remaining four doses that were administered throughout the demonstration project).

### Estimation of the cost of a fully immunized girl (FIG) and extrapolation to a one-year national program

In order to estimate the cost per FIG, we added up all two-year total costs in all the cost categories at all levels of the Ministries of Health and Education. We then divided these by the total number of girls who completed the dosing schedule of three and two vaccine doses in years one and two respectively. We calculated credibility intervals (CIs) around all estimated FIG costs [41] by determining upper and lower bound values for the model input parameters through an assumption of 20–30% positive and negative deviation from the baseline cost values, with adjustments to ensure value ranges were plausible. Subsequently maximum likelihood was used to obtain a gamma distribution for each parameter and then 10,000 Monte-Carlo simulations were run to generate possible random costs. The resulting cumulative totals were depicted in histograms and the 2.5 and 97.5 statistical percentiles were utilized to set the confidence intervals endpoints on the graphs.

We extrapolated the cost per FIG to a one-year program targeting all 10-year-old girls in Mozambique. For this projection, new FIG costs were estimated using model input parameters that excluded the first year third dose costs, to accommodate the current two-dose HPV vaccine schedule recommended by WHO. We also made estimates for a one-year program targeting all 10-year-old girls with just one dose because of the growing body of evidence for efficacy and effectiveness of only one-dose HPV vaccination creating the possibility of future dose reduction [42–44]). The target 10 year old girl population was calculated from the Mozambique 2007 census data from which the 2017 projected population was selected [45]. Values were categorized by province to simulate Mozambique’s immunization program costs calculation approach. No province straddles more than one of the three regions of the country and each HPV vaccine demonstration project district’s socioeconomic profile is similar to those of districts contained in the provinces found in each region.

All costs were collected in Mozambican Meticais and converted to US dollars using 2014 official exchange rates [46]. Analyses were conducted in Microsoft Excel 2017 (Microsoft, Redmond, WA, USA) and R software version 3.5.32019 (Vienna, Austria) [47]. We used the SQUIRE checklist when writing our report [48].

## Results

Forty-five personnel at the health facility, district, and national levels participated in the study, 27 from the Ministry of Health, six from the Ministry of Education, four from NGOs, three from multilateral agencies, three from research institutes, one from a bilateral organization, and one from a pharmaceutical company.

A summary of the number of health facilities, schools, and target population of eligible girls in each district is shown in Table 1.

**Table 1:** Schools and health facilities in the HPV vaccine demonstration districts

	Manhiça	Manica	Mocímboa da Praia
Health Facilities	13	20	7
Primary Schools	90	114	50
Necessary Teams	21	28	13
Target population Year 1	3350	3952	1254
Fully immunized girl year 1^a^	73.3%	47%	16%
Target population year 2	3588	4095	1452
Fully immunized girl year 2^a^	77%	51%	21%

^a^Coverage in percentage

The total implementation cost for the project in the three pilot districts was $523,602, 99.4% of which were direct medical costs. Note that costs for Manhiça district included Gavi subsidy funding while costs for the other two districts did not. In Table 2 the costs of implementing the HPV vaccine demonstration project activities are presented as total costs categorised into annual costs. Year one total project implementation costs were significantly higher than year two costs. The largest proportion in year one (23.8%) was personnel costs, while in year two most spending was on procurement (34%).

**Table 2:** Year one versus year two project costs

	Year 1	Year 2	Total
Direct Medical			
Cold Chain	$2000	$2000	$4000
Education Staff	$29,993	$15,826	$45,819
M&E	$56,050	$35,350	$91,400
Personnel	$77,543	$21,324	$98,867
Procurement^a^	$58,387	$67,387	$125,774
Social Mobilization	$33,500	$24,500	$58,000
Vaccination Campaign	$66,975	$29,767	$96,742
Direct Non-Medical			
Overhead	$1500	$1500	$3000
Grand Total	$325,948	$197,654	$523,602

^a^Procurement includes costs for acquiring vaccines and vaccine injection supplies

When costs were categorized by amounts spent in project locations including the central level and the three districts, the largest proportion were incurred in Manhiça accounting for 36.3% of total costs. Costs expended in Manica also amounted to a large proportion at 31.% while those spent at the central level and Mocimboa da Praia were the least and amounted to just 16.7% at each of the locations. At the central level, the largest implementation cost spending was on M&E activities, which accounted for 52% of the total costs, while procurement costs were the larger cost components in the districts accounting for 27, 34 and 27% in Manhiça, Manica da Mocimboa da Praia respectively.

The cost per FIG was $72 (CI: $62 - $83) in year one, $38 (CI: $37 - $40) in year two and $54 (CI: $49 - $61) for the whole project period. The cost of a FIG in each of the project districts in year 1, year 2, and whole project period were: Manhiça: $53 (CI: $41 - $63), $33 (CI: $29 - $43) and $42 (CI: $38 - $47); Manica: $66 (CI: $55 - $74), $33 (CI: $22 - $46) and $49 (CI: $40 - $54) and Mocimboa da Praia $373 (CI: $252 - $427), $123 (CI: $79 - $225) and $222 (CI: $136 - $361).

The cost of a FIG when the first year third dose implementation costs were excluded were: Year 1: $60 (CI: $50 - $72), Year 2: $38 (CI: $31 - $46) and for the whole two-year period $48 (CI: $42 - $55). When three of the total five doses that were given during the two-year period are excluded, and eligible girls each receive only one dose, the cost per FIG was; year one $30 (CI: $27 - $33)), year two $19 (CI:$15–$23) and year three $24 (CI:$22–$27).

The projected estimated costs for a 1 year two-dose and one-dose programs targeting all 10-year-old girls in Mozambique are presented in Table 3.

**Table 3:** Projected costs for a 1 year two- and one-dose HPV vaccination program

Province	Annual Total	
	Two doses	One dose
Maputo Provincia	$1,164,263 ($1,017,345 - $1,330,448)	$579,482 ($521,438 - $642,825)
Maputo Cidade	$660,112 ($576,813 - $754,336)	$328,554 ($295,644 - $364,468)
Gaza	$1,030,906 ($900,816 - $1,178,056)	$513,107 ($461,711 - $569,195)
Inhambane	$1,149,800 ($1,004,707 - $1,313,921)	$572,283 ($514,960 - $634,840)
Sofala	$1,533,068 ($1,339,611 - $1,751,897)	$763,046 ($686,614 - $846,454)
Manica	$1,495,776 ($1,307,024 - $1,709,281)	$744,484 ($669,912 - $825,864)
Zambezia	$3,482,485 ($3,043,032 - $3,979,571)	$1,733,318 ($1,559,698 - $1,922,787)
Tete	$1,894,574 ($1,655,498 - $2,165,003)	$942,976 ($848,521 - $1,046,052)
Nampula	$3,362,016 ($2,937,765 - $3,841,906)	$1,673,357 ($1,505,743 - $1,856,272)
Niassa	$1,111,315 ($971,078 - $1,269,942)	$553,128 ($497,723 - $613,590)
Cabo Delgado	$1,272,236 ($1,111,693 - $1,453,834)	$633,223 ($569,795 - $702,441)
Grand Total	$18,156,549 ($15,865,384 - $20,748,196)	$9,036,959 ($8,131,760 - $10,024,789)

## Discussion

This study presents the resource use and costs of the Mozambique HPV vaccine demonstration project. The implementation strategy of the project from planning and preparation to project execution was used to guide the evaluation. The results show that important cost determinants were vaccine price, number of administered doses, program startup costs and the necessity for rigorous demand creation. Additionally, the cost per FIG is relatively high at 40–70% of per capita health expenditure [49] and is higher in the district with worse socio-economic conditions, fewer eligible girls and schools. A similar, primarily school-based national HPV vaccination program in Mozambique, would be a costly undertaking, however fewer doses could lead to a substantial reduction in costs.

All category-specific costs except those of procurement of vaccines and vaccine injection supplies were lower in year two of the project, with the increase in the category’s cost explained by the larger total number of girls who were vaccinated in the second year relative to the first year. HPV vaccine’s price per dose of $4.60 (excluding shipping costs) is significantly higher than the price per dose of older vaccines such as Bacillus Calmette–Guérin (BCG) at $0.11 and measles at $0.24. Moreover, its price per dose is even higher than recently introduced pneumococcal conjugate and rotavirus vaccines dose prices of $3.50 and $2.55 respectively [50]. The high vaccine cost in our study is consistent with findings from other studies which have found that vaccine costs are a major component of total HPV vaccination programs costs [51–53]. Given that vaccine procurement (including injection supplies) costs often make up 50% of total national immunization programs’ vaccination costs [54], the findings from the current study raise concerns on the sustainability of HPV vaccines for Mozambique and other LMICs. Global level strategies to lower HPV vaccine pricing are needed to facilitate the vaccine’s adoption in these settings.

Personnel and demand creation costs were other major contributors to the total project costs. Both decreased significantly during the second year due to reduction in vaccine dosing from three to two doses, resulting in a week’s reduction of demand creation activities. Personnel costs accounted for 59% of total costs and consisted mainly of transportation costs and incentives for health workers to travel to schools to administer vaccines. These findings are important in the context of a potential future single dose HPV vaccination regimen [42–44] which would significantly reduce transportation and health worker allowance costs. Lower vaccine dosing (one- and two-dose schedules) will make the vaccine’s introduction more feasible in Mozambique.

Higher year one costs for personnel, social mobilization, and M&E reflect start-up costs that are required when initiating a program. Due to economies of scale, these start-up costs are expected to remain the same or rise modestly in order to cover a larger number of girls in a national scale up. In addition, social mobilization costs were higher in the district with the highest vaccination coverage, meaning that if resources are available, such costs should be considered as an important investment to stimulate increased vaccine uptake. Our findings are consistent with those of a study from Rwanda, the first country to roll out the HPV vaccine nationally in Africa [55].

The high cost per FIG of $54, observed in this study is consistent with costs per FIG found in other costing studies conducted within the context of HPV vaccine demonstration projects in LMICs [31–38]. Such expenditure on an individual recipient of the vaccine would amount to more than 50% of Mozambique’s current estimated health spending per capita of $92 [49]. The cost per FIG for the northern district was extremely elevated at $222 compared to $42.06 and $48.59 in for each of the other two districts. This is consistent with incremental implementation costs associated with the inefficiency of delivering health interventions in complex settings [2]. Our previously published process evaluation findings from this pilot project, demonstrated that distance and poverty were barriers to HPV vaccine delivery and uptake in the northern district. In this district approximately 50% of girls were not enrolled in schools and therefore, could not be reached with a school based vaccine delivery model [56]. National scale up of HPV vaccination in Mozambique will require an allowance for equity whereby poorer districts will need to be allocated higher funding to overcome the aforementioned barriers related to access. Moreover, immunization methods for increasing vaccination coverage in hard to reach populations, such as those recommended by WHO should be employed [57]. When the cost per FIG in the current study was extrapolated to a one-year countrywide two-dose program, the estimated total program cost of $18.2 m ($15.9 m - $20.7 m) was found to be relatively higher compared to annual costs for recently introduced new vaccines in Mozambique. Specifically, pneumococcal conjugate vaccine total program costs are $11 m while those for the rotavirus vaccine program are $16 m for cohorts of boys and girls while HPV vaccine would cover girls only. Nevertheless, program costs for a one-dose HPV vaccine would amount to half $9 m ($8 m - $10 m) the costs of a two-dose program, a value that is comparable to the cost of recently introduced national vaccination programs.

The results of this study should be interpreted in the context of parameter data limitations. While most cost inputs in this study were obtained from observational data, some inputs were based on health worker and teacher interviews. Specifically, time spent at schools by health workers was estimated (not directly measured) as was time spent by teachers organizing the HPV vaccination process in schools and observing vaccinated girls for adverse effects post immunization. Wages for both teachers and health workers were based on central level salary scales rather than actual district-level salaries. Fuel cost calculations did not consider distances but rather used uniform estimates based on money provided to all districts irrespective of number of schools or health facilities. A flat local to dollar currency exchange rate that did not account for inflation fluctuations that may have occurred during the period of the demonstration project was assumed in the study. Although these limitations may have led to both over- and under-estimation biases in the costs estimated, such biases are reflected in uncertainty estimates.

## Conclusion

Program implementation is complex and cost is a key factor in successful implementation. This study provides a framework that other researchers might use to identify, measure and value costs of implementing HPV vaccine delivery strategies. Additionally, the results provide important cost information that may be utilized by decision makers at Mozambique’s Ministry of Health. Introducing the HPV vaccine in Mozambique will be a costly endeavor however cost reduction may be achieved through adoption of a one-dose vaccination schedule. Given the cost drivers, the country will need to consider cost saving strategies including negotiation of vaccine price and the potential for economics of scale to mitigate demand creation costs so that the national roll out of HPV vaccine can be both feasible and sustainable.

## Supplementary information

**Additional file 1.** Key informant interview guide.

## Data Availability

The datasets used and/or analyzed during the current study are available from the corresponding author on reasonable request.

## Abbreviations

AEFI: Adverse Effects After Immunization
BCG: Bacillus Calmette–Guérin
CI: Credibility Intervals
cMYP: Comprehensive Multiyear Plan
FIG: Fully Immunized Girl
HPV: Human Papillomavirus
ICC: Invasive Cervical Carcinoma
IEC: Information Education and Communication
KI: Key Informant
KIIs: Key Informant Interviews
LMICs: Low and Middle-Income Countries
M&E: Monitoring and Evaluation
MOH: Ministry of Health
NGO: Non-Governmental Organization
NIP: National Immunization Program
WHO: World Health Organization
