# Supplementary material for: Implementation strategy and cost of Mozambique’s HPV vaccine demonstration project
Source: BMC Public Health. 2019 Oct 29;19:1406. doi: 10.1186/s12889-019-7793-y (PMC6819423; doi:10.1186/s12889-019-7793-y)
Supplement: Supplementary file 1 — Additional file 1. Key informant interview guide. [file 12889_2019_7793_MOESM1_ESM.docx]

**Key Informant Interview Guide 2014**

**Introduction**

My name is ______________________ . I am part of the team implementing the GAVI Full Country Evaluation (FCE) in Mozambique. The team is composed of staff from the Universidade Eduardo Mondlane (UEM) and Health Alliance International (HAI). The overall aims of the FCE are to **identify and evaluate processes, networks, and systems that influence vaccine coverage.**

The main topics I would like to address in today’s interview are about the HPV vaccine demonstration project taking place in Manhiça, Manica and Mocímboa da Praia.

**Please, go through the consent process and obtain the participant’s signature.**

Record the participant’s details and organizational level:

| 1. Institution |  |
| --- | --- |
| 1. Occupation |  |
| 1. Position |  |
| 1. How long has the participant been in this position |  |

**Audio recording starts here!**

**Questions**

1. **Decision-making:** When and how was the decision to implement the demonstration project in the country made? What were the decision-making steps and who were the main actors in making that decision? Was any scientific evidence used to make the decision? What considerations were taken to decide on the timing (when) and where to implement the project? We noticed that the initial proposal was for Mozambique to implement the project in three districts but GAVI suggested implementation in only one district – what were the reasons for the decision?
2. **Proposal development and submission:** Which team developed the proposal? Who led and coordinated it? Were there specific tasks for each team member? For instance, what was your role and/your organizations role in the process? Were the Gavi document that you had to fill in easy to understand? Was the proposal submitted within the deadline? Were there any challenges during the proposal submission process? For example, delays? Did GAVI provide any technical support during the process?
3. **Design of the demonstration project:** one of the key decisions taken in the proposal that was submitted to GAVI was the selection of the target group, which is 10-year-old girls (instead of 9,11, 12 or 13-year-old girls). What was the rationales for selecting this specific target group? The HPV proposal stated that the vaccine would be administered in schools and communities. Were other sites, such as health facilities, considered?
4. **Technical support:** We know that CISM (Manhiça Center for Health Research) and FDC (Fundação para o Desenvolvimento da Comunidade) have specific roles in the implementation process of the demonstration project. How was the decision about their roles made? What are the roles of other partners such as UNICEF, WHO and VillageReach? Later the INS (Instituto Nacional de Saúde) was included in the technical working group. Do you know how the role of the INS was decided upon? Was UNFP asked to provide support?
5. **GAVI funds:** How was the budget developed? For instance, was a specific consultant hired to develop the budget? This time, GAVI funds to the country were disbursed early (6 months prior to the launch of the Demonstration) project. Do you know what facilitated the process this time (compared to when the pneumococcal vaccine (PCV) was introduced)? Why did the transfer of funds from MOH [Ministry of Health] to CISM and FDC take so long? Why did the transfer of funds to Manhiça district take so long? In the proposed budget submitted to GAVI, UNICEF was expected to provide $6500 for social mobilization. How was that done? Was it through a transfer of funds to MOH? In the proposed budget submitted to GAVI, WHO was expected to provide $5000? How have these funds been disbursed to the MOH?
6. **Inclusion of more districts:** When was the decision to include Manica and Mocímboa da Praia in the demonstration project made? How was that decision made (in consultation with stakeholders or was it a MOH decision)? Did the later inclusion of Mocímboa da Praia and Manica, in the demonstration project, influence the planning, budget, and disbursement of funds? Was there a contingency plan in place, in case the funds were not enough to conduct all the activities in the three districts?
7. **Launch date:** In January the plan was to launch the project on April 7. However, the date was changed to 12 May. When was the decision to postpone to another date made? And, why 12 May? How did the national immunization program influence the launch date change?
8. **Census:** Why was the decision to conduct a target girls census made much later (two and a half months before the project launch and not during proposal development)?
9. **Supply and Logistics**
   1. How were the quantities of vaccines needed estimated? When did the vaccines for Manhiça arrive in country? Was the plan to distribute the vaccine a week before launching the project? If not, why did it happen only a few days before project launch?
   2. Was the cold chain ready to receive the vaccines in the districts? Did all refrigerators (at all health facilities) have HPV vaccine related stickers?
   3. Was a readiness assessment to receive the vaccine conducted? Were the results shared with everyone? Was the readiness confirmation process well-received?
   4. Have the second and third dose vaccines arrived in the country? Why didn’t these second batch of vaccines arrive in country before the launch date?
   5. Did the the NIP logistical personnel receive any HPV vaccine specific training?
   6. How was the daily monitoring of refrigerators’ temperatures (from the central warehouse in Zimpeto down to the health facility and mobile outreach levels) ensured (especially during weekends and public holidays)?
10. Regarding **training** of health workers and teachers, which funds were used to develop and print training materials? In Manhiça, we know that this was done in a cascade manner, starting with teacher trainers who then trained the rest of the teachers in the district. Is there anything that that did not happen as planned? Can you comment on it?
    1. When did the trainings for health workers and teachers in the other districts supported by the government (Manica and Mocímboa da Praia) take place? How many health workers and teachers were trained? Who trained them? How was the quality assurance of the training done?
    2. Were health workers and teachers from all three districts trained on monitoring of adverse events? if yes, when?
11. **M&E**: When know that the tools were not available on time. When exactly did they become ready? Who was responsible for developing/updating the tools? Were the tools tested? What caused the delay in printing the M&E tools? Were the tools for monitoring of adverse effects developed, printed/copied and distributed on time? Was there a plan B in case the tools were not ready on time? What was it?
12. **Creating Adequate Demand**: What were the reasons for delays in broadcasting messages through radio spots until a week before the project launch? If there were delays related to funding, what exactly were the reasons and what was the source?
13. **Implementation**: Was there any challenge with the denominators used for the first dose? What challenges were those, and how will they be overcome in the next doses? Is there a contingency plan if the target is not reached? Is a week enough to vaccinate all children, as anticipated? Registration issues – were there many problems? Why? Data flow?
14. We would like to know if MOH and partners anticipate the need to integrate, in the future, HPV vaccination with other health services for adolescents in Mozambique (for instance, Geração Biz/SAAJ**)**? If yes, what is being done or has been planned?
15. We are about to finish: in your perspective, how do you think GAVI can improve the way it has been supporting Mozambique in the preparation process for the implementation of the HPV demonstration process from proposal development to launch?
16. To finish: recently there was a change in the head of the NIP. Do you think that affected the HPV launch program? In terms of the frequency of planning meetings and decision-making, etc.? Consequences of that transition? Did the change of the head of NIP impact the way information was provided to technical working group members (negatively or positively)?
17. How did you (/your organization) join the HPV technical working group? What challenges did you face and what facilitated that process?
18. How was your interaction with the stakeholders such as office of the first lady, CISM, UNICEF, WHO, INS, FDC?
19. **Funds**: We know that when the GAVI funds were disbursed, MOH had enough funds for all activities in Manhiça, Manica and Mocímboa da Praia. Why did it take so long for the funds to be transferred to CISM and FDC? And to Manhiça district? Did MOH have to co-finance activities in Manhiça district? How much was provided to Manica and Mocímboa da Praia? Were the budget lines the same as those of Manhiça district? Were all implementation needs covered in all the districts?
20. The launch date was postponed from 14 to 16. Why did that happen?

**Key Informant Interview Guide 2015**

1. Regarding the HPV demonstration project in the (Manhiça/Manica or Mocímboa da Praia) district:
   1. Can you make a comparison of the implementation of the project last year and this year?
   2. What challenges did you have this year for each of the following categories?
      1. Teacher, community leader and health personnel training,
      2. Logistics and cold chain,
      3. M&E tools
      4. Social mobilization
      5. School based vaccination
      6. Community outreach vaccination
   3. What do you think should be improved in future HPV vaccinations?
   4. For national roll out
      1. Where do you think the vaccine should be offered to girls?
      2. If it will continue to be in schools what do you think should be improved?
